# Lamotrigine and Lithium Combination for Treatment of Rapid Cycling Bipolar Disorder: Results From Meta-Analysis

**DOI:** 10.3389/fpsyt.2022.913051

**Published:** 2022-07-14

**Authors:** Gao Zhihan, Sun Fengli, Lv Wangqiang, Shen Dong, Jin Weidong

**Affiliations:** ^1^Department of Clinical Psychology, Hangzhou First Hospital, Hangzhou, China; ^2^Department of Psychiatry, Zhejiang Province Mental Health Center, Hangzhou, China; ^3^Department of Psychiatry, Jinhua Second Hospital, Jinhua, China; ^4^Department of Psychiatry, Jiaxing Kangci Hospital, Jiaxing, China; ^5^Department of Psychiatry, Zhejiang Chinese Medical University, Hangzhou, China

**Keywords:** lamotrigine, lithium, combination therapy, RCBD, bipolar disorder

## Abstract

**Objective:**

The objective of this study is to observe the effect of combination of lithium and lamotrigine in treatment of rapid-cycling bipolar disorder (RCBD).

**Method:**

We searched MEDLINE, EMBASE, Cochrane Library in English and CBM, CNKI, WANFANG, and CSSCI in Chinese to find literature from 1 January 2000 to 31 December 2020 related to the combination of lithium carbonate and lamotrigine for treatment of RCBD.

**Results:**

Five comparison studies with 265 subjects of 131 cases in a study group and 134 cases in a control group met the inclusion criteria and were included for the final meta-analysis. The comprehensive analysis shows that the study group had a significant lower score in mental symptoms than the control group (*Z* = 2.34, *P* = 0.02) with a random model (*X*^2^ = 33.02, *df* = 7, *P* < 0.01). However, the differences were only shown in PANSS (*Z* = 5.18, *P* < 0.01) and BPRS (*Z* = 3.08, *P* < 0.01). There was no difference in response rate (54.9 vs. 45.7%; OR = 1.47; 95% CI: 0.79~2.73; *Z* = 1.21, *P* > 0.05,) and remission rate (47.9 vs. 45.9%; OR = 1.05; 95% CI: 0.49~2.25; *Z* = 0.13, *P* > 0.05,) found between the two groups. The response rate of lamotrigine and lithium combination was significantly higher compare to that of monotherapy of lithium in patients with no treatment resistant (82 vs. 54%; OR = 4.26; 95% CI: 1.65~10.99; *Z* = 3.99, *P* < 0.01) with the fixed effect model (*X*^2^ = 0.89, *df* = 1, *P* > 0.05, *I*^2^ = 0%).

**Conclusion:**

The combination of lithium and lamotrigine resulted in better improvement of psychotic symptoms and higher response rate in patients with RCBP with no treatment resistant.

## Background

The long-term course of bipolar disorder is typified by recurring mood episodes of opposite polarity and mixed states. Rapid-cycling bipolar disorder (RCBD) refers to the presence of at least 4 mood episodes in previous 12 months that meet the criteria for manic, hypomanic, or major depressive episode (1). RCBD also includes ultra-rapid (cycle lengths of days to weeks, including 48-h cycling) and ultra-ultra-rapid cycling (cycle lengths of up to 24 h) according to cycling speed (2–4). However, the course is not clear, and antidepressants maybe an extra induced course (2, 5). RCBD has been estimated to affect approximately 20% of patients with bipolar disorder (6). Patients with RCBD are more likely to demonstrate non-response to traditional mood stabilizers and have poorer prognosis and increased risk for suicide compared to those without RCBD (7). Moreover, frequent comorbidities with substance use disorders pose an additional negative impact on treatment outcomes of patients with RCBD, including greater risk for treatment non-adherence, more hospitalizations and mood episodes, lower rates of remission (8), and decreased quality of life (6). Therefore, most guidelines for treatment of RCBD suggest a combination of mood stabilizers and stopping and prohibiting usage of antidepressants.

Lamotrigine is a mood stabilizer, it also is a first line drug in the acute and maintenance treatment of bipolar disorder, and only one drug called “mood stabilizer for depression”. Lamotrigine is often used in treatment of BD due to more common depressive symptom (9). A meta-analysis summarized lamotrigine's effectiveness and safety in unipolar and bipolar depression and found that lamotrigine outperformed placebo regarding depressive symptoms (studies = 11, *n* = 713 vs. *n* = 696; SMD = −0.15; 95% CI = −0.27, −0.02, *p* = 0.02, heterogeneity: *p* = 0.24) and response (after removing one extreme outlier; RR = 1.42; 95% CI = 1.13–1.78; *p* = 0.003; heterogeneity: *p* = 0.08). Conversely, lamotrigine did not differ regarding efficacy in depressive symptoms, response, or remission from lithium, olanzapine + fluoxetine, citalopram, or inositol (studies = 6, *n* = 306 vs. *n* = 318, *p*-values =0.85–0.92) (10). Therefore, lamotrigine was superior to placebo in improving unipolar and bipolar depressive symptoms without causing more frequent adverse effects/discontinuations and did not differ from lithium, olanzapine + fluoxetine, citalopram, or inositol. Lamotrigine have better effectiveness in treatment of bipolar depression.

Lithium is a first-line option in acute and maintenance treatments of bipolar disorder and the only one drug that can prevent suicide, because there is high suicidal risk among individuals affected by BD. However, this is not the only rationale behind lithium's use in bipolar disorder. Lithium of is the choice of treatment for this disorder, with special emphasis on pharmacology, and it has effectiveness in both depression and mania. Alternatives should be potent mood stabilizers such as monotherapy to avoid polypharmacy (9). However, the fact is that polypharmacy in bipolar treatment are more often, especially for RCBD.

The concept of double mood stabilizers has been suggested for treatment of bipolar disorder (8, 11, 12). The clinical therapeutic effect of double mood stabilizer is better than that only one mood stabilizer for patients with bipolar disorder and less interaction between drugs. However, the combination of lithium and valproate was more common than that of lithium and lamortigine. However, lamotrigine is called "mood stabilizer for depression" and can decrease the switch to mania induced by antidepressants (9, 13), and it may further strengthen lithium's ability to stabilize mood. It also improve depression symptoms which are less likely to respond to treatment with lithium alone or divalproex. A study also showed that lamotrigine is superior to placebo in treatment of RCBD (14). A case report on addition of lamotrigine to valproic acid had a successful outcome in a case of rapid-cycling bipolar affective disorder (15). Many guidelines suggest that anticonvulsant drugs and mood stabilizers such as valproate and carbamazepine have a good effect on treatment of RCBD. But in fact, the treatment of RCBD is often by combination therapy, such as combination of valproate and an atypical antipsychotic drug or the combination of dual mood stabilizers, such as valproate and lithium carbonate. While this way may be better for mania, but not be effective for depression in RCBD. Therefore, it may be a good choice to select lithium carbonate, which is effective for mania, and lamotrigine, which is effective for depression. Therefore, their combination may play a role in bipolar disorder, especially in RCBD. This study was a meta-analysis on the combination of lamotrigine and lithium used for treatment of RCBD.

## Methods

### Literature Retrieval Methods

This study was performed according to the recommendations of MOOSE (16). Two reviewers independently searched databases. Chinese databases included Chinese Biomedical Database (CBM), China National Knowledge Infrastructure (CNKI), WANFANG, and Chinese Social Sciences Citation Index (VIP). The English databases included MEDLINE, EMBASE, and the Cochrane Library.

### Search Keywords: Lamotrigine, Lithium, Bipolar Disorder, Rapid Cycling

**Figure 1:**
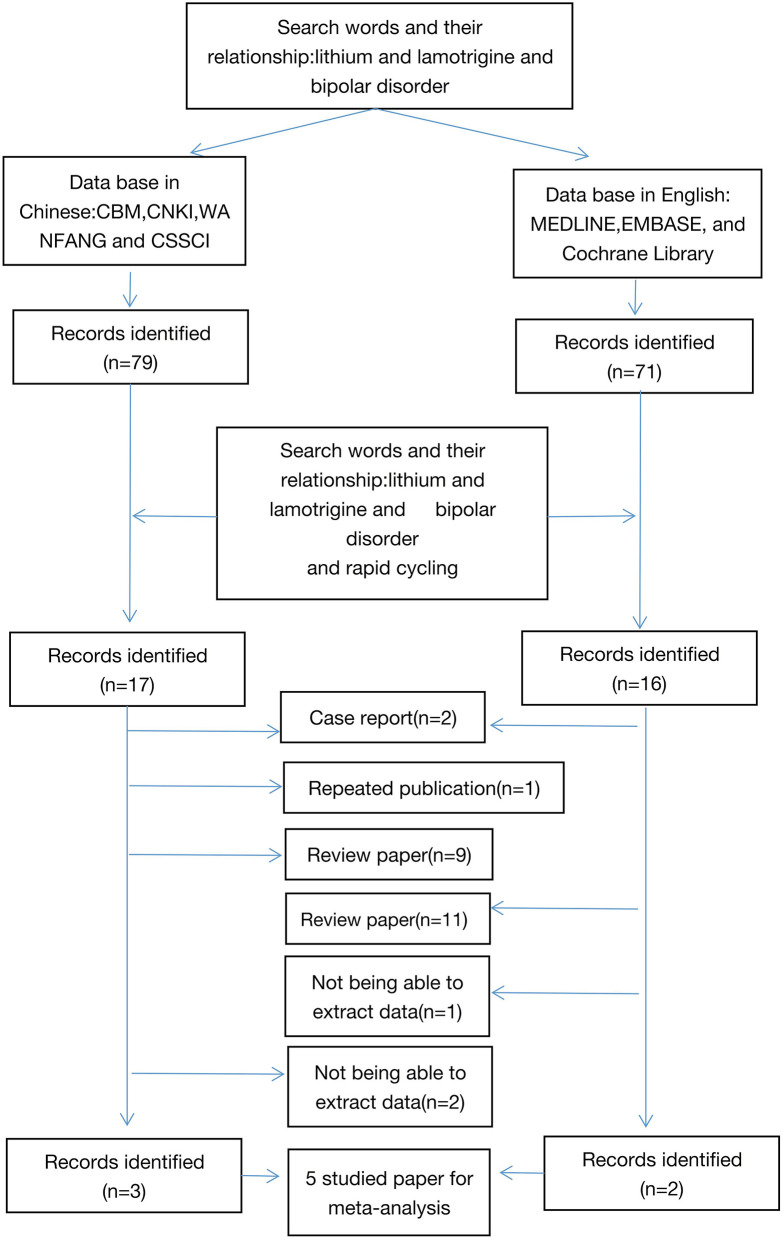
Flowchart of selection of studies for inclusion in meta-analysis.

The search strategy was based on combinations. To retrieve all articles, we searched articles using “Bipolar index and bipolar disorder (or mood disorder or mania or bipolar depression or depression,” and then were further screened the articles related. The retrieval literature was published from January 1, 2000 to January 1, 2021. References of retrieved articles were cross-searched to identify any studies missed by the electronic search strategies. Dr. GZH completed the literature retrieval and participated in the collection of data (Figure 1).

### Inclusion and Exclusion Criteria

The two researchers reviewed the initial retrieved publications independently. Discrepancy was resolved by discussion by all reviewers. Studies that met the following criteria were those whose design was combination of lamotrigine and lithium compared to only lithium. However, articles that had incomplete or unidentified data were excluded, as well as abstracts, reviews, case reports, letters, and duplicate publications.

Two psychiatrists reviewed each included article independently using the 11-item checklist that was recommended by the Agency for Healthcare Research and Quality (AHRQ) (17). The following information was extracted: first author, publication time, sample size, study population, assessment tools, and index of therapeutic effects (Table 1) (6, 7, 18–20). Dr. GZH, Dr. LWQ, and Dr. SFL assessed the quality of the researched article according to the AHRQ.

**Table 1:** Characteristics of studies included in the meta-analysis.

**Reference**	**Study design**	**Experimental group (EG) cases**	**Drugs of EG**	**Control group (CG) cases**	**Drugs of CG**	**Quality score**	**Index for therapeutic effect**
Chen et al. (18)	RCT	20	Lithium + lamotrigine	20	lithium	8	Response rate PANSS, BPRS
Wang et al. (6)	RCT	18	Lithium + Valproate + lamotrigine	18	Lithium + Valproate	8	Response rate Remission rate YMRS, MADRS CGI
Kemp et al. (7)	RCT	23	Lithium + Valproate + lamotrigine	26	Lithium + Valproate + Placebo	8	Response rate Remission rate YMRS,MADRS CGI
Cai (19)	RCT	30	Lithium + lamotrigine	30	lithium	8	Response rate Remission rate
Liu and Han (20)	RCT	40	Lithium + lamotrigine	40	lithium	8	PANSS, BPRS

*PANSS, positive and negative symptom scale; BPRS, Brief Psychotic Rating Scale; YMRS, Yung Manic Rating Scale; MADRS, Montgomery-Asberg Depression Rating Scale; CGI, Clinical Global Impression; EG, Experimental group (Lamotrigine + lithium); CG, Control group (Lithium)*.

### Statistical Analysis

All statistical analyses were performed using the Statistical Analysis System software (Revman 5.2). For the overall effect, the *P* value less than 0.05 by two-tailed test was considered statistically significant. The heterogeneity of all the involved studies was assessed with *I*^2^. When it was lower than 50%, studies with an acceptable heterogeneity were considered, and then the fixed-effects model with Mantel-Haenszel method was used; otherwise, a random effect model with the Der Simonian and Laird (DL) method was adopted. Dr. SD and Dr. SFL completed the statistical analyses. The main index included response rate, remission rate, and symptom change.

Assessment of publication bias was investigated for each of the pooled study groups mainly by Egger's linear regression test. As a supplement approach, Begg's rank correlation was also applied to assess for potential publication bias.

## Results

### Study Characteristics

Five comparison studies with 265 subjects of 131 cases in a study group and 134 cases in a control group that met the inclusion criteria were included for the final meta-analysis. The five studies consist of 3 in Chinese and 2 in English [5, 25~38]. The sample size of the studies ranged from 18 to 40. The assessment tools for therapeutic effectiveness in the studies are listed as follows: PANSS, BPRS, YMRS, MARDS, and CGI. The main features of the 5 articles are summarized in Table 1. AHRQ scores suggested that all the 5 studies scored 8 and had high quality.

### Comparison of Mental Symptoms Between the Study Group and the Control Group

**Figure 2:**
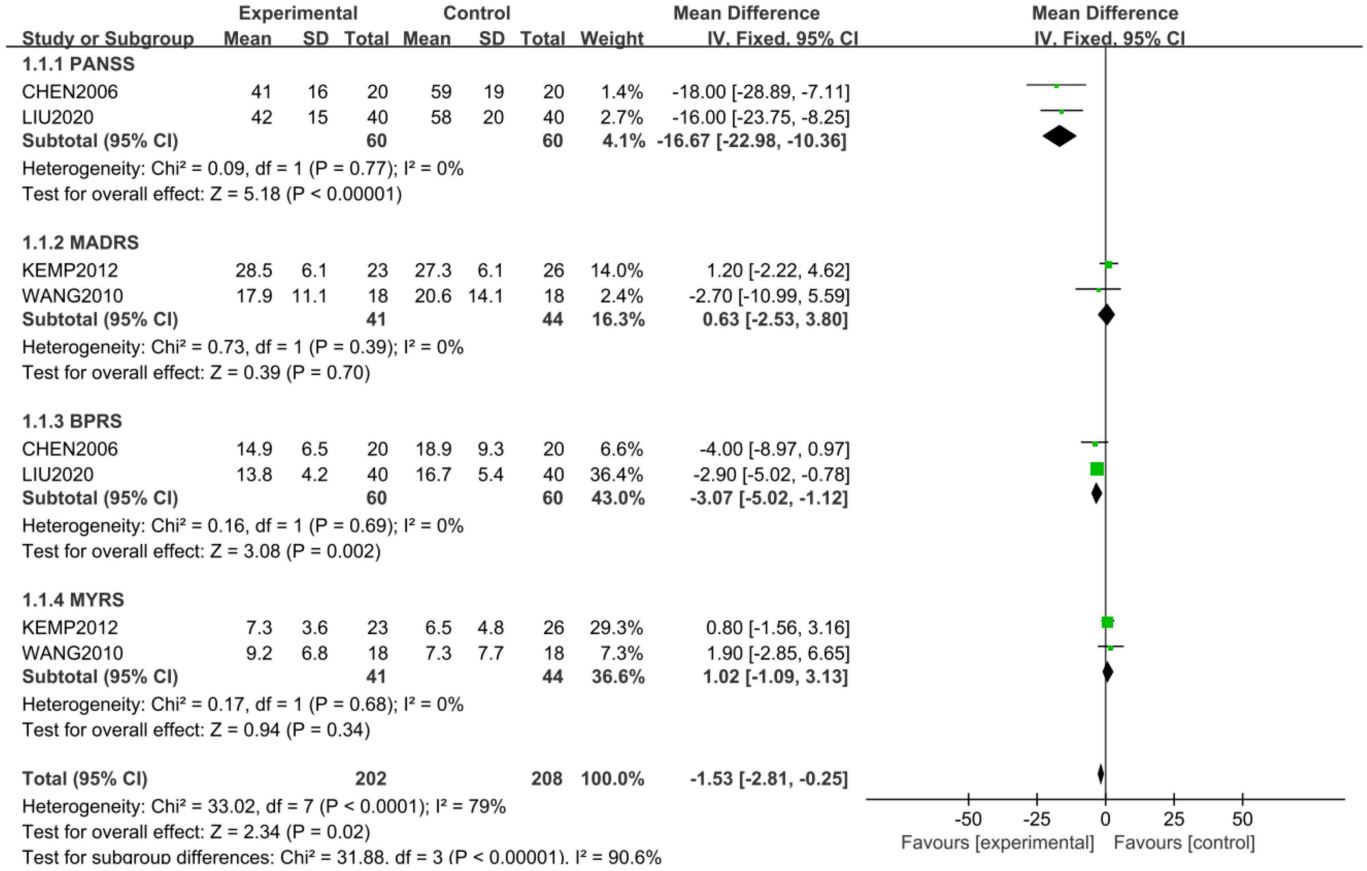
Comparison of different mental symptoms between the study and control groups. The comprehensive analysis shows that the study group had significantly lower score in mental symptoms than the control group (*Z* = 2.34, *P* = 0.02) with a random-effects model (*X2* = 33.02, *df* = 7, *P* < 0.01). However, the differences were only shown in PANSS (Z = 5.18, *P* < 0.01) and BPRS (Z = 3.08, *P* < 0.01), and not in MADRS (*Z* = 0.39, *P* > 0.05) and YMRS (Z = 0.94, *P* > 0.05).

The scale assessment for mental symptom during treatment was used in 4 studies. PANSS and BPRS were used in 2 studies, and YMRS and MARDS were used in the other studies. A subgroup analysis was conducted for mental symptoms because of the different way of assessment. The comprehensive analysis showed that the study group had a significantly lower score in mental symptoms than the control group (*Z* = 2.34, *P* = 0.02) with the random model (*X*^2^ = 33.02, *df* = 7, *P* < 0.01). However, the differences were only shown in PANSS (*Z* = 5.18, *P* < 0.01) and BPRS (*Z* = 3.08, *P* < 0.01), and were not shown in MADRS (*Z* = 0.39, *P* > 0.05) and YMRS (*Z* = 0.94, *P* > 0.05) (Figure 2). However, publishing bias was found by funnel plot analysis.

### Comparison of Response and Remission Between Study Group and the Control Group

**Figure 3:**
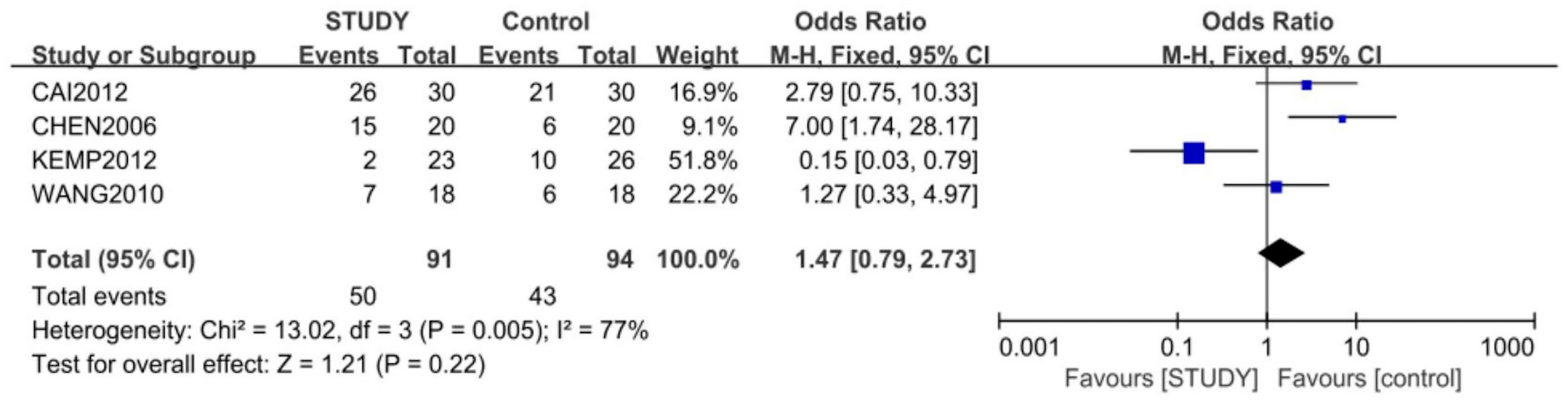
Comparison of response rate between the study and control groups. The random-effects model was used for this analysis (*X*^2^ = 13.02, *df* = 3, *P* < 0.01, *I2* = 77%). No difference in response rate was found between the two groups (OR = 1.47; 95% CI: 0.79~2.73; *Z* = 1.21; *P* > 0.05).

Four of the 5 studies, with 185 subjects, were included for the meta-analysis of response rate, which was 54.9% in study group and 45.7% in control group. The random-effects model was used for this analysis (*X*^2^ = 13.02, *df* = 3, *P* < 0.01, *I*^2^ = 77%). No difference in response rate was found between the two groups (OR = 1.47; 95% CI: 0.79~2.73; *Z* = 1.21; *P* > 0.05,) (Figure 3), but the small publishing bias was not found by the funnel plot analysis.

**Figure 4:**
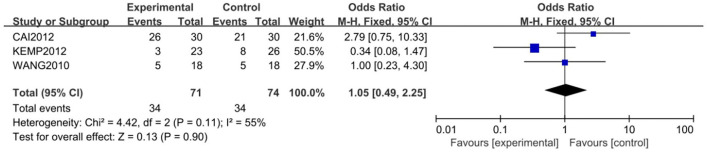
Comparison of remission rate between the study and control groups. Three of the 5 studies, with 145 subjects, were included for the meta-analysis of remission rate, which was 47.9% in the study group and 45.9% in the control group. The random-effects model was used for this analysis (*X*^2^ = 4.42, *df* = 2, *P* > 0.05, *I2* = 55%). No difference in remission rate was found between the two groups (OR = 1.05; 95% CI: 0.49~2.25; Z = 0.13, *P* > 0.05).

Three of the 5 studies, with 145 subjects, were included for the meta-analysis of remission rate, which was 47.9% in the study group and 45.9% in the control group. The fixed-effects model was used for this analysis (*X*^2^ = 4.42, *df* = 2, *P* > 0.05, *I*^2^ = 55%). No difference in response rate was found between the two groups (OR = 1.05; 95% CI: 0.49~2.25; *Z* = 0.13, *P* > 0.05,) (Figure 4). Some publishing bias was found by the funnel plot analysis.

**Figure 5:**
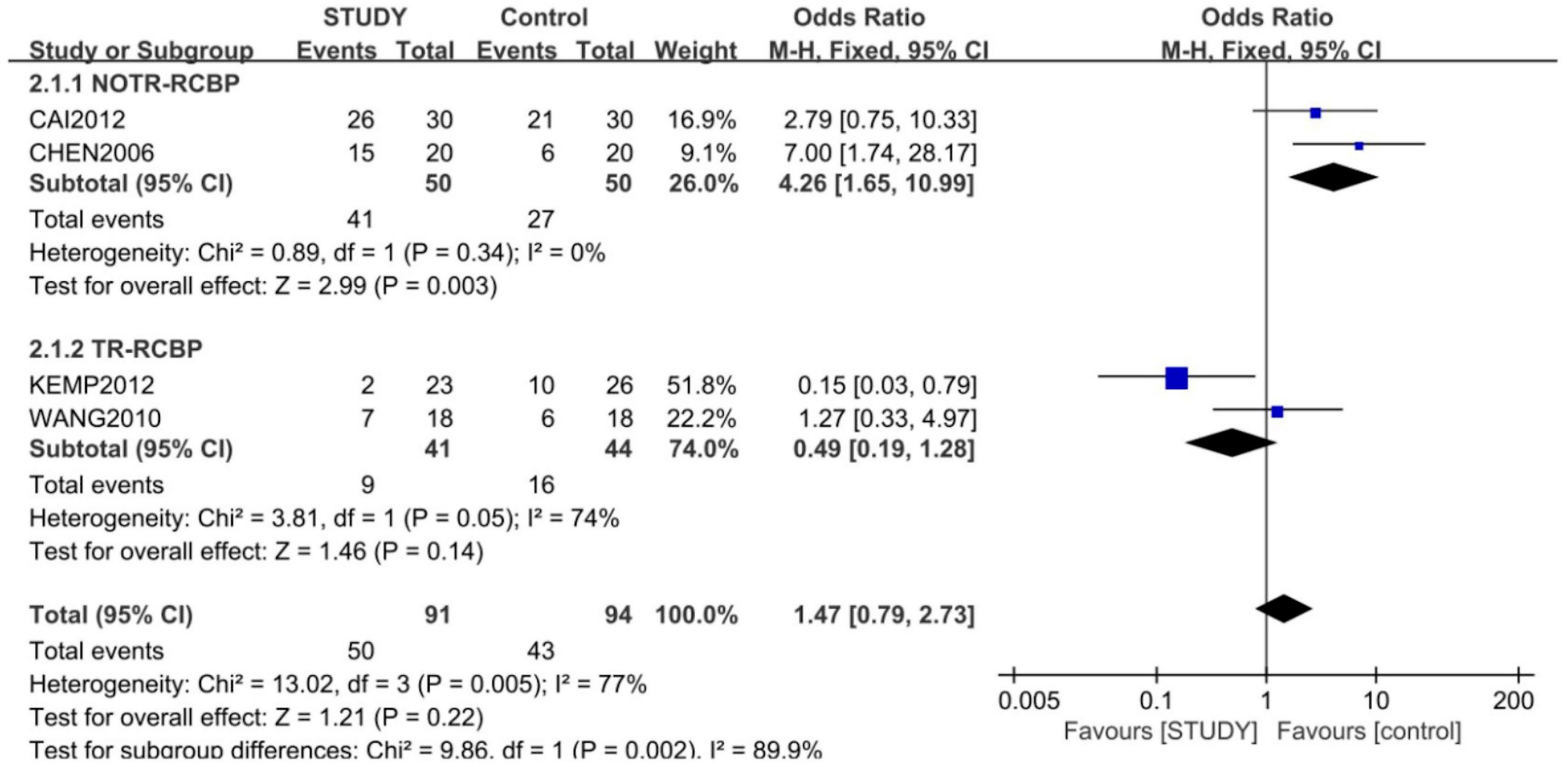
Subgroup analysis of response between the study and control groups. A subgroup meta-analysis for response rate was also conducted according to treatment-resistant or not. The response rate of the lamotrigine and lithium combination was significant higher than that of the monotherapy of lithium in patients with no treatment resistance (82 vs. 54%;OR = 4.26; 95% CI: 1.65~10.99; *Z* = 3.99; *P* < 0.01) with the fixed-effects model (*X*^2^ = 0.89, *df* = 1, *P* > 0.05, *I*^2^ = 0%), but the response rate of lamotrigine, lithium, and valprorate combination was not significantly higher than that of placebo, lithium, and valprorate combination in patients with treatment resistance (21.9 vs. 36.3%; OR = 0.49; 95% CI: 0.0.19~1.28; *Z* = 1.46; *P* > 0.05), with the random-effects model (*X*^2^ = 3.81, *df* = 1, *P* = 0.05, *I*^2^ = 77%).

A subgroup meta-analysis for response rate was also conducted according to treatment resistant or not. The response rate of lamotrigine and lithium combination was significantly higher than that of monotherapy of lithium in patients with no treatment resistant (82 vs. 54%; OR = 4.26; 95% CI: 1.65~10.99; Z = 3.99; *P* < 0.01) with the fixed-effects model (*X*^2^ = 0.89, *df* = 1, *P* > 0.05, *I*^2^ = 0%). However, the response rate of lamotrigine, lithium, and valprorate combination was not significantly higher than that of the placebo, lithium, and valprorate combination in patients with treatment resistance (21.9 vs. 36.3%; OR = 0.49; 95% CI: 0.19~1.28; *Z* = 1.46, *P* > 0.05), with the random-effects model (*X*^2^ = 3.81, *df* =, *P* = 0.05, *I*^2^ = 77%) (Figure 5).

## Discussion

Rapid-cycling bipolar disorder represents a frequent severe subtype of illness that has been associated with poor response to pharmacological treatment. To our knowledge, this is the first meta-analysis of the lamotrigine and lithium combination in treatment of RCBD. This meta-analysis included two randomized, parallel-group, placebo-controlled trials in English that evaluated the efficacy of a triple medication combination in RCBD (6, 7) and three randomized controlled trials in Chinese (18–20). The two studies in English are evaluating the therapeutic effect of lamotrigine in combination with lithium and divalproex in patients with treatment-resistant RCBD not co-occurring substance use disorder.

Four of the 5 studies, with 185 subjects, were included for the meta-analysis of response rate, which was 54.9% in the study group and 45.7% in the control group. Three of the 5 studies, with 145 subjects, were included for the meta-analysis of remission rate, which was 47.9% in the study group and 45.9% in the control group. There was no difference in both response and remission. However, the lamotrigine and lithium combination a play role in more improvement of mental symptoms, especially of psychotic symptoms, rather than depressive and manic symptoms compared to the control group. However, subgroup meta-analysis showed that the lamotrigine and lithium combination had higher response rate than the monotherapy of lithium in patients with no TR-RCBD.

Controlled studies on treatment of RCBD indeed are few. The search returned 206 articles, and ultimately 25 were selected for review (21). Only six randomized controlled trials specifically designed to study a rapid cycling population were found. Most data were derived from *post-hoc* analyses of trials that included rapid cyclers. The literature find that (i) most patients with rapid cycling perform worse in the follow-up period, (ii) lithium has efficacy comparable to that of anticonvulsants, (iii) there is inconclusive evidence on the comparative acute or prophylactic efficacy of the combination of anticonvulsants vs. anticonvulsant monotherapy, (iv) antipsychotics such as aripiprazole, olanzapine, and quetiapine are effective against acute bipolar episodes, (v) olanzapine and quetiapine appear to be equally effective to anticonvulsants during acute treatment, (vi) aripiprazole and olanzapine appear promising for the maintenance of response of rapid cyclers, and (vii) presence of rapid cycling might be associated with antidepressant use. According to opinion above, atypical antipsychotics maybe a relatively better selection for treatment of RCBD, although the lamotrigine and lithium combination shows better improvement in mental symptoms and higher response in patients without TR-RCBD. Other therapeutics were also useful selection such as vagus nerve stimulation (VNS) (22) and levothyroxine augmentation therapy (23).

According to monotherapy, lamotrigine is similar to lithium in treatment of patients with RCBD in a small sample trial (24). Also, according to monotherapy, both lamotrigine and lithium were superior to placebo at prolonging the time to intervention for any mood episode (lamotrigine vs. placebo, *P* = 0.02; lithium vs. placebo, *P* = 0.006). Lamotrigine was superior to placebo at prolonging the time to a depressive episode (*P* = 0.02). Lithium was superior to placebo at prolonging the time to a manic, hypomanic, or mixed episode (*P* = 0.006) (25). It is obvious that studies on the combination of lamotrigine and lithium in treatment of RCBD should be carried out. It was a pity there were few clinical trials on the combination of lamotrigine and lithium in treatment of RCBP. Up to now, only the 5 study trials were found. Therefore, this meta-analysis was seen as a supplement of the few trials. In fact, the combination therapy was proved to more effective than monotherapy of lithium for RCBD.

This study had several limitations. First, the sample size of this meta-analysis was relatively small. Only 5 studies and 265 subjects were involved. Second, collecting data style may influence the result of investigation, i.e., different criteria of RCBD can get different response. Different response and remission rates were found between no TR and TR patients. Third, the dose and level of drugs in the blood was not focused,because the effect of lituium are closely related to drug level in blood (26), fourth, side effects related to the drugs, especially to the combination therapy of lamotrigine and lithium, were not included. Fifth, not all the studies had blind observation. These factors are partly responsible for the source of pool response and remission rate of the study also affecting the authors to see the real significance of the combination. The next step should be designed as a multi-center, double-blind comparative study to observe the effectiveness of the lamotrigine and lithium combination in treatment of RCBD.

## Data Availability Statement

The original contributions presented in the study are included in the article/Supplementary Material, further inquiries can be directed to the corresponding author.

## Author Contributions

GZ participated in the collection of data and the writing of the article. GZ, LW, and SF assessed the quality of the researched articles. SD and SF completed most of the statistic analyses. JW and SF participated in the design, statistical processing, and final revision of the article. All authors reviewed and researched the whole article and read and approved the manuscript.

## Conflict of Interest

The authors declare that the research was conducted in the absence of any commercial or financial relationships that could be construed as a potential conflict of interest.

## Publisher's Note

All claims expressed in this article are solely those of the authors and do not necessarily represent those of their affiliated organizations, or those of the publisher, the editors and the reviewers. Any product that may be evaluated in this article, or claim that may be made by its manufacturer, is not guaranteed or endorsed by the publisher.

## Abbreviations

EMBASE: Excerpta Medical Database
CBM: Chinese Biomedical Database
CNKI: China National Knowledge Infrastructure
CSSCI: Chinese Social Sciences Citation Index (VIP)
YMRS: Yung Manic Rating Scale
BRMS: Beck-Rafaelsdn Mania Rating Scale
SPSS: Statistic Product and Service Solutions
PANSS: positive and negative symptom scale
BPRS: Brief Psychotic Rating Scale
TR: treatment-resistant
RR: relative risk
BD: bipolar disorder
CI: confidence interval
MADRS: Montgomery-Asberg Depression Rating Scale
CGI: Clinical Global Impression.
