# Supplementary material for: Lamotrigine and Lithium Combination for Treatment of Rapid Cycling Bipolar Disorder: Results From Meta-Analysis
Source: Front Psychiatry. 2022 Jul 14;13:913051. doi: 10.3389/fpsyt.2022.913051 (PMC9329592; doi:10.3389/fpsyt.2022.913051)
Supplement: Supplementary file 1 [file Table_1.DOCX]

**Identification of studies from Chinese and English databases and registers**

Records removed *before screening*:

Duplicate records removed (n =1)

Records marked as ineligible by automation tools (n =4 )

Records removed for other reasons (n = 5)

Records identified from*:

Chinese Databases (n =34 )

Registers (n = 0)

**Identification**

Records screened

(n =24 )

Records excluded criteria

(n = 11)

Reports sought for retrieval

(n =13)

Reports not retrieved

(n =2 )

**Screening**

Reports assessed for eligibility

(n =11 )

Reports excluded:

Case report (n =3 )

Review (n =1 )

Biological study (n =2)

Reports of included studies

(n =5)

**Included**
